# DNA Damage Reduces the Quality, but Not the Quantity of Human Papillomavirus 16 E1 and E2 DNA Replication

**DOI:** 10.3390/v8060175

**Published:** 2016-06-22

**Authors:** Molly L. Bristol, Xu Wang, Nathan W. Smith, Minkyeong P. Son, Michael R. Evans, Iain M. Morgan

**Affiliations:** 1 VCU Philips Institute for Oral Health Research, Department of Oral and Craniofacial Molecular Biology, Virginia Commonwealth University School of Dentistry, Richmond, VA 23298, USA; xwang@vcu.edu (X.W.); smithnw2@mymail.vcu.edu (N.W.S.); paekm@vcu.edu (M.P.S.); evansmr2@vcu.edu (M.R.E.); 2 VCU Massey Cancer Center, Richmond, VA 23298, USA

**Keywords:** papillomaviruses, cancer, replication, etoposide, integration, E1, E2, TopBP1, HPV

## Abstract

Human papillomaviruses (HPVs) are causative agents in almost all cervical carcinomas. HPVs are also causative agents in head and neck cancer, the cases of which are increasing rapidly. Viral replication activates the DNA damage response (DDR) pathway; associated proteins are recruited to replication foci, and this pathway may serve to allow for viral genome amplification. Likewise, HPV genome double-strand breaks (DSBs) could be produced during replication and could lead to linearization and viral integration. Many studies have shown that viral integration into the host genome results in unregulated expression of the viral oncogenes, E6 and E7, promoting HPV-induced carcinogenesis. Previously, we have demonstrated that DNA-damaging agents, such as etoposide, or knocking down viral replication partner proteins, such as topoisomerase II β binding protein I (TopBP1), does not reduce the level of DNA replication. Here, we investigated whether these treatments alter the quality of DNA replication by HPV16 E1 and E2. We confirm that knockdown of TopBP1 or treatment with etoposide does not reduce total levels of E1/E2-mediated DNA replication; however, the quality of replication is significantly reduced. The results demonstrate that E1 and E2 continue to replicate under genomically-stressed conditions and that this replication is mutagenic. This mutagenesis would promote the formation of substrates for integration of the viral genome into that of the host, a hallmark of cervical cancer.

## 1. Introduction

Human papillomaviruses (HPVs) are small, double-stranded DNA viruses that cause a variety of human diseases, including, but not limited to, cervical cancer and head and neck cancers [1,2]. Moreover, HPV16 is the most prevalent genotype in both cervical (around 50% of cases) and head and neck cancers (around 90% of the HPV-positive cases) [2]. Key risk factors for carcinogenesis include viral persistence and viral DNA integration [3,4,5,6,7].

Papillomaviruses replicate their genomes utilizing two proteins; E2, a DNA-binding protein that binds to the origin of replication, then recruits E1, a viral helicase that opens and unwinds DNA and initiates DNA replication in association with cellular factors [8,9,10,11,12,13]. E1 and E2 require interactions with host DNA replication and repair (DDR) proteins to carry out viral replication [8,9,10,14,15,16,17,18,19,20,21,22]. HPV replication has the potential to introduce DNA double-stranded breaks (DSB) into the viral genome that would result in the production of substrates for the integration of HPV genomes into that of the host [14,16,18,23]. Integration of the viral genome is thought to confer selective advantages to the host cell and is detected in nearly 90% of cervical carcinomas, but is much less understood in head and neck cancer [4,23,24,25,26,27,28]. Moreover, it is thought that integration allows for unregulated expression of the viral oncogenes E6 and E7, allowing for cellular immortalization, unregulated proliferation and increased genomic instability, all contributing to carcinogenesis [6,28,29,30].

Here, we investigated the quality of DNA replication under conditions of genomic stress. Our previous work demonstrated that the presence of etoposide, even though it induced cell cycle arrest, had no effect on the levels of DNA replication mediated by E1 and E2 [31]. Similarly, when we remove topoisomerase II β binding protein I (TopBP1) from the cell using short hairpin RNA (shRNA), we do not decrease E1–E2-mediated DNA replication, even though we destroy the DNA replication foci containing E1, E2, TopBP1, bromodomain containing 4 (Brd4) and a host of other replication and repair factors [32]. To investigate the quality of replication, we replicated an HPV16 origin-containing plasmid that contained the *lacZ* gene [33]. Following the rescue of the E1–E2 replicated plasmids, the levels of DNA replication were determined using real-time PCR [34]. The results demonstrate that elimination of TopBP1 or treatment of cells with etoposide did not affect the levels of DNA replication as reported previously [31,32], but dramatically increased the mutation frequency. Elimination of TopBP1, as well as causing decreased fidelity of E1–E2 DNA replication, promoted catastrophic damage to the host genome following host DNA replication demonstrating the essential nature of the TopBP1 protein. Overall, the results demonstrate that E1 and E2 proteins have a remarkable capacity to replicate under conditions of genomic stress and that this replication is error prone under such conditions. This error-prone replication would promote double-stranded DNA breaks in the viral genome that would provide substrates for the integration of the viral genome into that of the host. Our results suggest that any therapy targeting HPV DNA replication should also be investigated for the effect on the quality of that replication. This can be done using the simple blue-white screen employed in this report.

## 2. Materials and Methods

### 2.1. Cell Culture

C33a cells were obtained from ATCC (Manassas, VA, USA) and grown in Dulbecco’s Modified Eagle’s Medium (Invitrogen, Carlsbad, CA, USA) supplemented with 10% fetal bovine serum and were routinely passaged every 3–4 days and routinely monitored for mycoplasma.

### 2.2. Plasmids

All of the HPV16 plasmids utilized in these studies have been previously used and described by this laboratory: HPV16 pOriLacZ (pOriLacZ) [33], HPV16 E1-(hemagglutinin, HA) (E1) [17], HPV16 E2 [35], pSuper control shRNA and shRNA TopBP1 [32].

### 2.3. Transient DNA Replication Assay

C33a cells were plated out at 6 × 10^5^ in 100-mm dishes. The following day, plasmid DNA was transfected using the calcium phosphate method [36]. Three days post-transfection, low molecular weight DNA was extracted using the Hirt method as previously described [37]. The digested sample was extracted twice with phenol:chloroform:isoamyl alcohol (25:24:1) and precipitated with ethanol. Following centrifugation, the DNA pellet was washed with 70% ethanol, dried and resuspended in a total of 150 µL water (this DNA was also used for DNA mutagenesis analysis, described below). Forty two microliters of sample were digested with DpnI (New England Biolabs, Ipswitch, MA, USA) overnight to remove unreplicated pOri16LacZ; the sample was then digested with ExoIII (New England Biolabs) for 1 h. Replication was determined by real-time PCR, as described previously [34].

### 2.4. DNA Mutagenesis Analysis

As previously described [33], 42 µL of sample were digested overnight with DpnI to remove unreplicated pOri16LacZ. The sample was then extracted once with phenol:chloroform:isoamyl alcohol (25:24:1), precipitated with ethanol and washed with 70% ethanol. The DNA was re-suspended in 150 µL of 10% glycerol; 75 µL were electroporated into DH10B bacteria and plated onto kanamycin Lysogeny Broth (LB) agar containing 100 µg/mL X-gal (Genesee Scientific, San Diego, CA, USA). DH10B carrying pOri16LacZ with a wild-type LacZ are blue, and those with mutations in LacZ are light blue/white. Plasmid DNA was prepared using the Qiaprep spin miniprep kit (Qiagen, Hilden, Germany), and samples were eluted in 50 µL water. Five microliters of DNA were digested with BamHI and then subjected to 1% agarose gel electrophoresis to highlight plasmid rearrangements.

### 2.5. Western Blots

Cells were trypsinized, washed twice with phosphate-buffered saline (PBS), pelleted, then re-suspended in 200 μL of lysis buffer (0.5% Nonidet P-40, 50 mM Tris, pH 7.8, 150 mM NaCl) supplemented with a protease inhibitor mixture (Roche Molecular Biochemicals, Indianapolis, IN, USA). The cell and lysis buffer mixture was incubated on ice for 30 min, centrifuged for 10 min at 18,000× *g* at 4 °C, and the supernatant was collected. Protein levels were determined utilizing the Bio-Rad protein assay (Bio-Rad, Hercules, CA, USA). Equal amounts of protein were boiled in 4× Laemmli sample buffer (Bio-Rad). Samples were then loaded onto a 4%–12% gradient gel (Invitrogen), ran at 120 V for ~2 h and transferred at 40 V overnight onto nitrocellulose membranes (Bio-Rad) using the wet blot method. The membrane was then blocked in Odyssey blocking buffer (diluted 1:1 with PBS), at room temperature for 1 h. After blocking, the membrane was probed with noted antibodies diluted in blocking buffer and incubated overnight at 4 °C: E1(HA) rabbit 1:1000 (Abcam ab9110, Cambridge, MA, USA), E2-TVG261 mouse 1:1000 (Abcam ab 17185), p-histone H2A.X rabbit 1:1000 (Cell Signaling #9718S, Danvers, MA, USA), β-actin mouse 1:2000 (Santa Cruz sc-81178, Dallas, TX, USA), Chk2 rabbit 1:1000 (Cell Signaling #2662S), p-Chk2 (T68) rabbit 1:1000 (Cell Signaling #2661S), TopBP1 mouse 1:1000 (Bethyl; A300-111A, Montgomery, TX, USA). Following incubation with primary antibody, the membrane was washed with 0.01% PBS-Tween wash buffer before probing with corresponding Odyssey secondary antibody diluted 1:20,000, goat anti-mouse IRdye 800CW or goat anti-rabbit IRdye 680CW for one hour at room temperature. The membrane was then washed in 0.01% PBS-Tween before infrared scanning using the Odyssey Li-Cor imaging system.

### 2.6. Fluorescence-Activated Cell Sorting Analysis

Cell cycle analysis was examined using propidium iodide staining to determine DNA content. The cells were trypsinized, washed twice with PBS and centrifuged at 500 × *g* for 5 min. Cells were then re-suspended in 0.5 mL PBS, and 10 mL 75% ethanol were added slowly while vortexing to prevent cell clumping. The cells were left in fixative at 4 °C for 30 min, centrifuged at 200 × *g* for 5 min and washed once with PBS supplemented with 1% bovine serum albumin (BSA). Cells were then suspended in fresh propidium iodide (Sigma, St. Louis, MO, USA)/RNase A (Qiagen) solution and incubated at 37 °C for 30 min. Samples were immediately analyzed by flow cytometry (Guava Technologies, Millipore, Billerica, MA, USA).

### 2.7. Etoposide Treatment

Varying doses of etoposide were added to adherent C33a cells (500 nM, 1 µM, 5 µM, 10 µM, 25 µM, 100 µM) (Figure S1). Forty-eight hours after the addition of etoposide, cells were harvested and analyzed via fluorescence-activated cell sorting (FACS). The dose of 5 µM resulted in marginal apoptosis along with correct cell cycle arrest and was used for all subsequent studies.

### 2.8. Small interfering RNA (siRNA) and Metaphase Spread

C33a cells were plated out at 6 × 10^5^ in 100-mm dishes. The following day, cells were transfected with 1 µg siRNA luciferase control or 1 µg siRNA TopBP1 (Ambion custom select siRNA; siRNA luc sense C.G.U.A.C.G.C.G.G.A.A.U.A.C.U.U.C.G.A.dT.dT, antisense U.C.G.A.A.G.U.A.U.U.C.C.G.C. G.U.A.C.G.dT.dT; siRNA TopBP1 sense G.U.G.G.U.U.G.U.A.A.C.A.G.C.G.C.A.U.C.dT.dT, antisense G.A.U.G.C.G.C.U.G.U.U.A.C.A.A.C.C.A.C.dT.dT) utilizing the lipofectamine RNAiMax protocol (Invitrogen 13778-100). Sixteen hours post-transfection, cells were washed. Zero-point-one micrograms per milliliter of colcemid were supplemented for 16 h, and cells were then harvested; cells were washed twice with 1 × Hank’s balanced salt solution and then trypsinized. The pellet was re-suspended in 200 µL residual supernatant, and 5 mL of pre-warmed 75 mM KCl were slowly added. Cells were incubated at 37 °C for 5 min. Five microliters of freshly-prepared Carnoy’s fixative (3:1 methanol:glacial acetic acid) were gently added and mixed by gently inverting the 15-mL conical flask. This mixture was centrifuged at 200 × *g* for 5 min. Supernatant was poured out and the pellet resuspended in 10 mL fixative. This was repeated five times. Twelve microliters of the final resuspension were added dropwise to glass slides and allowed to dry in a humidified chamber. Dried slides were allowed to age for a week at room temperature, and slides were stained with 4′,6-diamidino-2-phenylindole (DAPI, Santa Cruz sc-3598, Dallas, TX, USA); coverslips were mounted using Vectashield mounting medium (ThermoFisher NC9265087, Rockford, IL, USA). Images were collected with a Zeiss LSM700 confocal microscope configured around an AxioImager and analyzed using Zen lite software (ZEN black/blue, Carl Zeiss Microscopy, Thornwood, NY, USA).

### 2.9. Chromatin Immunoprecipitation

A 100-mm^2^ dish of 60% confluent C33a cells was transfected with 1 μg of pOriLacZ, 1 μg of E1, 1 µg of E2 and 1 µg pSuper control shRNA or 1 µg pSuper shRNA TopBP1, using the calcium phosphate method [36]. The following day, cells were washed twice with PBS and transferred to 15-cm^2^ dishes; at this time, if noted, 5 µM etoposide were added. At 48 h post-transfection, cells were cross-linked with 1% formaldehyde at room temperature for 15 min. The cross-linking reaction was stopped using 0.125 M glycine. Cells were harvested and lysed, and chromatin was sheared utilizing the method previously published by Gauson et al., 2015 [32]. The chromatin concentration was measured using a NanoDrop spectrophotometer, and 100 μg of chromatin were used per antibody experiment. The antibodies used were as follows, per immunoprecipitation (IP): 10 μL of sheep anti-HPV16 E2 (amino acids 1–201) prepared and purified by Dundee Cell Products (Dundee, United Kingdom); 2 μg of rabbit anti-HA for detecting E1(HA) (Abcam; ab9110); 2 μg of mouse anti-TopBP1 (Bethyl; A300-111A). The antibodies and chromatin were incubated along with 20 μL of slurry of IgG protein A/IgG protein G A/G magnetic beads (ThermoFisher Scientific; 26162). The chromatin, bead and antibody slurry was incubated with rotation at 4 °C overnight. The following day, beads were washed and chromatin prepared, as previously described [32]. TaqMan qPCR using the pOri primer and probe set was used to quantify the levels of E2, E1 and TopBP1 at the HPV origin of replication.

### 2.10. Immunofluorescence

C33A cells were grown on coverslips to 40%–45% confluence and transfected using the Lipofectamine 2000 reagent (Life Technologies 1166809, Carlsbad, CA, USA). At 48 h post-transfection, the cells were fixed with pre-chilled (−20° C) methanol for 4 min and incubated at −20 °C, then washed three times with PBS. The cells were permeabilized by incubation with 0.2% Triton X-100 in PBS for 15 min at 4 °C and washed three times with PBS. The coverslips were blocked with 5% BSA in PBS for 1 h at room temperature and washed twice with PBS. Cells were then incubated with a dilution of the primary antibody E1(HA) rabbit 1:1000 (Abcam ab9110), in PBS overnight at 4 °C in a humidified chamber. The coverslips were washed three times with PBS and incubated with a 1:1000 dilution of Alexa Fluor 488 goat anti-rabbit secondary antibody (Life Technologies A11034) in PBS for 1 h, followed by three washes with PBS. Cellular DNA was stained with 0.1 µg/mL DAPI (Santa Cruz sc-3598) for 15 min at room temperature and washed three times with PBS. The coverslips were mounted using Vectashield mounting medium (ThermoFisher NC9265087) and then sealed. Images were acquired using a Zeiss LSM 700 confocal laser scanning microscope configured around an AxioImager and analyzed using Zen lite software.

## 3. Results

### 3.1. shRNA Knockdown of TopBP1 is Mutagenic for HPV16 E1–E2-Mediated DNA Replication

**Figure 1:**
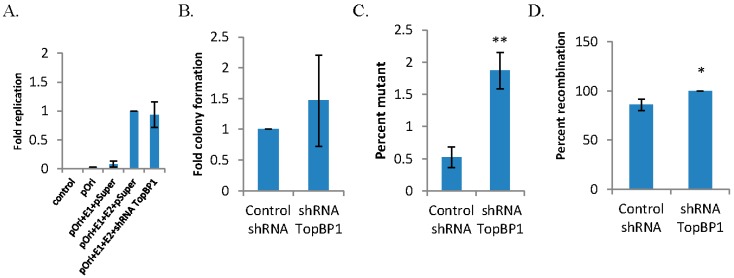
Graphic summaries of replication data following short hairpin RNA (shRNA) for topoisomerase II β binding protein I (TopBP1). (**A**) C33a cells were transfected with 1 µg pOriLacZ, 1 µg E1, 1 µg E2 and 1 µg control shRNA or 1 µg TopBP1 shRNA. Seventy two hours post-transfection, low molecular weight DNA was harvested, digested with DpnI and ExoIII, and the levels of pOriLacz were monitored via quantitative PCR (qPCR). Data are from three replicative experiments and presented as the fold replication of pOriLacZ. (**B**) Additional DNA was harvested and DpnI digested; DNA was then electroporated into DH10B *E. coli* and plated onto agar plates containing kanamycin and β-galactosidase. Data are presented as the fold of colony formation. (**C**) The total number of colonies were counted and compared to the total number of light blue/white colonies observed, and data are presented as the percent of mutation. (**D**) White colonies were then picked; plasmid DNA was prepared, digested with *Bam*HI and resolved on an agarose gel; data are presented as the percent of recombination events observed. * *p* < 0.05; ** *p* < 0.01.

We originally identified the interaction between the cellular replication and repair factor TopBP1 and HPV16 E2 [37]. Subsequently, we have demonstrated that TopBP1 interaction with E2 is essential for optimal DNA replication and for the viral life cycle and that TopBP1 is recruited into E1–E2 DNA replication foci [32,38]. shRNA targeting TopBP1 destroys these E1–E2 DNA replication foci while having no effect on the levels of replication [32]. This report investigated the quality of the DNA replication mediated by E1 and E2 in the absence of the large DNA replication foci by knocking down TopBP1 using shRNA as previously described [32,39]. As expected, shRNA against TopBP1 did not affect the levels of E1–E2-mediated DNA replication (Figure 1A). To measure the fidelity of replication, the replicated DNA used in Figure 1A was electroporated into DH10B *Escherichia coli* (*E. coli*) and scored for the number of blue and white colonies when the bacteria were plated onto agar containing kanamycin and X-gal. This experiment was carried out three times, and average results are shown with associated error bars in Figure 1A–D; total results are summarized in Table 1. TopBP1 shRNA did not alter overall *E. coli* total colony formation (Figure 1B); however, TopBP1 shRNA did significantly increase the incidence of mutations in the *lacZ* gene resulting in an increase in the percentage of white colonies (Figure 1C). To determine what types of mutations were present in the mutant colonies, we prepared plasmid DNA from the transformed bacteria, digested them with *Bam*HI, and resolved the DNA on an agarose gel. The predicted band size of 2.9 kb and 1.2 kb was not seen in most cases with control shRNA, consistent with the plasmids having a recombined phenotype, rather than a mutation in the *lacZ* gene. However, recombination was the only observed phenotype with shRNA TopBP1 (1D), suggesting an overall trend for increased recombination events.

**Figure 2:**
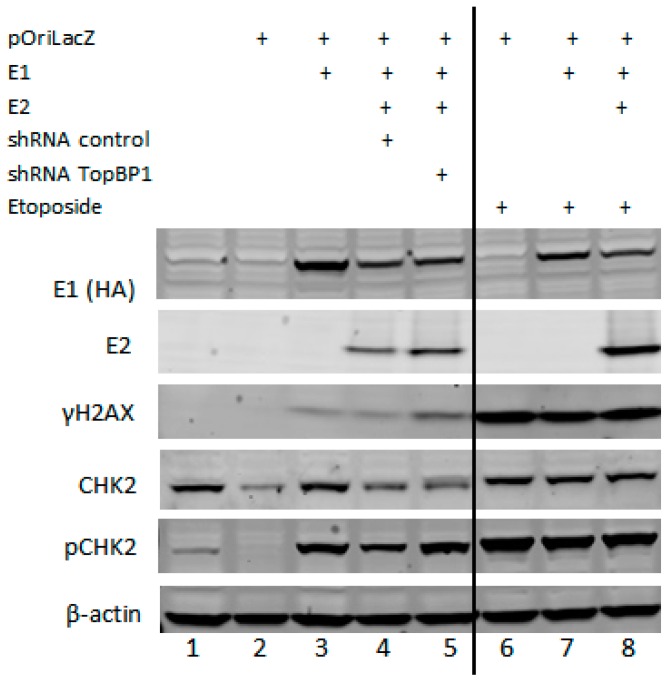
Western blots confirm the presence of viral replication proteins E1 and E2 along with DNA damage response proteins. C33a cells were transfected with 1 µg each of noted plasmids and harvested 72 h post-transfection for protein extract preparation. Lanes 6–8 also received 48 h of 5 µM etoposide. Protein extracts were Western blotted for the proteins indicated, and β-actin was used as a loading control.

As there were no alterations in replication levels, but increases in mutation frequency and recombination events, we asked whether alterations in viral proteins or DDR proteins were contributing factors. Western blot data presented in Figure 2 (densitometry provided in Table 2) demonstrate that TopBP1 shRNA does not alter E1 levels and increases E2 levels, as shown previously [39]. Expression of E1 alone is enough to initiate the DDR, as seen by increasing levels of γH2AX and increasing levels of p-Chk2, and TopBP1 shRNA enhances this response. The activation of the DDR by E1 has also been observed by others [22,40,41,42].

### 3.2. siRNA Knockout of TopBP1 Leads to Chromosome Dysfunction in Metaphase Cells

While we previously reported that the interaction between TopBP1 and E2 is essential for optimal viral DNA replication and for the viral life cycle, it is also known that TopBP1 is essential for replicating cells during the initiation of host DNA replication, as well as having a vital role in the DDR [43].

**Figure 3:**
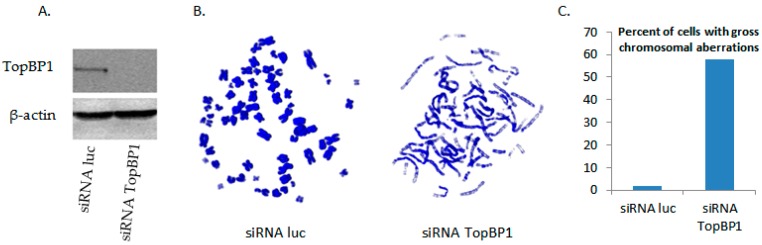
Loss of TopBP1 critically alters chromosomal phenotype. (**A**) C33a cells were transfected with 1 µg siRNA luciferase control or 1 µg siRNA TopBP1. Thirty two hours post-transfection, cells were harvested, and protein extract was prepared. Protein extracts were Western blotted for TopBP1, and β-actin was used as the loading control. (**B**) As in (A), C33a cells were transfected. Sixteen hours post-transfection, cells were washed; 0.1 µg/mL colcemid was supplemented for 16 h; and cells were then harvested and prepared for metaphase spread. Slides were stained with DAPI and imaged via confocal microscopy. The image on the left is a representative spread for siRNA luciferase control cells; the image on the right is the representative spread for siRNA TopBP1. (**C**) Metaphase spreads were quantified and presented as the percent of cells exhibiting gross chromosomal aberrations.

The cells transfected with the TopBP1 shRNA did not have a reduction in cell number, suggesting that the cells were replicating in the short term in the absence of TopBP1. As this knockdown was so damaging for E1–E2 DNA replication (Figure 1), we investigated the effects of siRNA TopBP1 knockout on cellular DNA via metaphase spreads. We chose to utilize siRNA for this experiment, because it maximizes the proportion of knockdown cells; C33a cells were transfected with siRNA luciferase as the control or with siRNA TopBP1, and Western blot data confirming efficient knockout are presented in Figure 3A. These cells were then supplemented with 0.1 µg/mL colcemid for 16 h and harvested for metaphase spreads; Figure 3B,C shows the representative images of these spreads, and Figure 3D quantifies the percentage of cells observed with gross chromosomal aberrations. We observed a dramatic increase in the percentage of TopBP1 siRNA cells that exhibited chromosomes that were elongated and/or segmented compared to control siRNA cells, demonstrating the vital role of TopBP1 for the integrity of host DNA. This, together with the data presented in Figure 1A–D, suggests that E1 and E2 have the capability to replicate even under conditions of extreme genomic stress and that this replication is error prone under such conditions. We propose that the virus utilizes a salvage replication pathway that the cell also employs that allows for DNA replication that is highly mutagenic.

### 3.3. The DNA Damaging Agent Etoposide Promotes Low Fidelity E1–E2 DNA Replication

**Figure 4:**
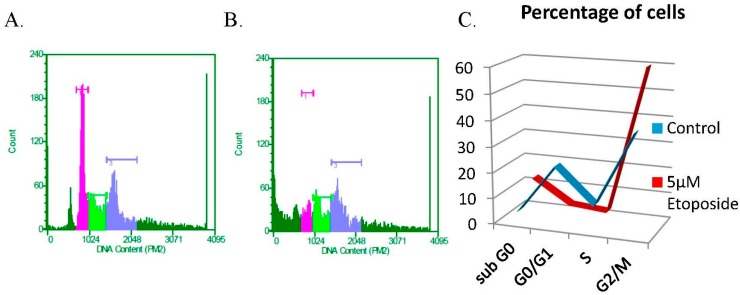
Treating C33a cells with five micromoles of etoposide results in G2/M arrest. C33a cells were plated; 5 µM etoposide were supplemented 24 h post-plating. Forty eight hours after the addition of etoposide, cells were harvested and analyzed via FACS. (**A**) Representative control cell cycle FACS analysis; (**B**) representative etoposide cell cycle FACS analysis; (**C**) data are presented as the percentage of cells observed in each phase of the cell cycle from triplicate experiments.

**Figure 5:**
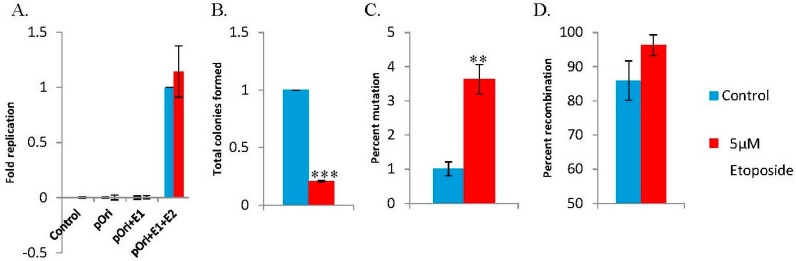
Graphic summaries of replication data following 5 µM etoposide. (**A**) C33a cells were transfected with 1 µg pOriLacZ, 1 µg E1 and 1 µg E2, in the presence or absence of 5 µM etoposide. Seventy two hours post-transfection, low molecular weight DNA was harvested, digested with DpnI and ExoIII, and the levels of pOriLacZ were monitored via qPCR. Data are from three replicative experiments and presented as the fold replication of pOriLacZ. (**B**) Additional DNA was harvested and DpnI digested; DNA was then electroporated into DH10B *E. coli* and plated onto agar plates containing kanamycin and β-galactosidase. Data are presented as the fold of colony formation. (**C**) The total number of colonies was counted and compared to the total number of light blue/white colonies observed, and data are presented as percent mutation. (**D**) White colonies were then picked, plasmid DNA was prepared, digested with *Bam*HI and resolved on an agarose gel; data are presented as the percent of recombination events observed. ** *p* < 0.001; *** *p* < 0.0001.

Our previous work showed that E1–E2 DNA replication levels are not altered by etoposide, even though the cell cycle is arrested, demonstrating the cell cycle independence of E1–E2 DNA replication [31]. To investigate the quality of this DNA replication, the blue-white assay was used. A dose response curve for etoposide on C33a cells was conducted (Figure S1), and for all subsequent studies, we chose to utilize 5 µM etoposide for 48 h (Figure 4). Etoposide did not affect the levels of E1–E2-mediated DNA replication in C33a cells (Figure 5A), as has been shown previously for 293T cells [31]. The replicated DNA used in Figure 5A was electroporated into DH10B *E. coli*; colonies were scored, and the average results are shown with associated error bars in Figure 5B–D, with total results summarized in Table 3. We observed that etoposide significantly reduced total colony formation (Figure 5B). While there were significantly less colonies, the percentage of mutation in the *lacZ* gene resulted in a dramatic increase in the percentage of white colonies (Figure 5C). The transformed bacteria in these mutant colonies were used to prepare plasmid DNA, which was then digested with *Bam*HI, and the DNA was resolved on an agarose gel. Similar to the data presented in Figure 1D, the predicted band sizes of 2.9 kb and 1.2 kb were not seen in most cases with control cells, consistent with the plasmids having a recombined phenotype, rather than a mutation in the *lacZ* gene. Again, etoposide increased the incidence of recombination events (Figure 5D).

**Figure 6:**
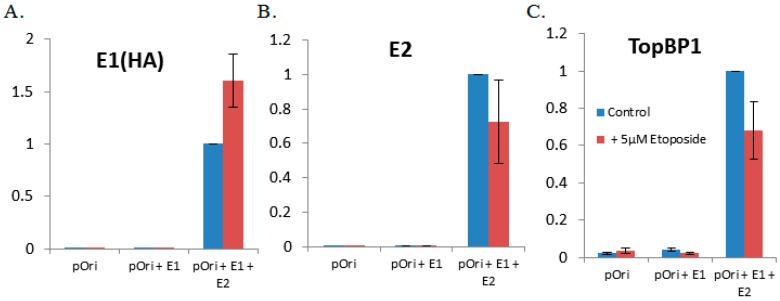
Etoposide does not significantly alter the ability of E1, E2 or TopBP1 to be recruited to the origin. C33a cells were transfected with 1 µg pOriLacZ, 1 µg E1 and 1 µg E2, in the presence or absence of 5 µM etoposide. Forty eight hours post-transfection, cells were cross-linked, harvested and lysed, and chromatin was sheared via sonication. One hundred micrograms of chromatin were used per antibody. The chromatin, bead and antibody slurry was incubated with rotation at 4 °C overnight. The following day, beads were washed and chromatin prepared. TaqMan qPCR using the pOri primer and probe set was used to quantify the levels of (**A**) E1(HA), (**B**) E2 and (**C**) TopBP1 at the HPV origin of replication. qPCR data are presented as the fold from three replicate experiments.

We also wanted to determine whether etoposide altered the expression levels of the viral replication proteins E1 or E2 and whether this drug was initiating the DDR. In Lanes 6–8 of the Western blot presented in Figure 2, it appears that etoposide has increased the expression level of E2 and has also enhanced the protein levels of both γH2AX and p-Chk2. We also wanted to determine whether etoposide influenced the recruitment of E1, E2 or TopBP1 to the viral origin. We utilized the ChIP assay to determine that there were no significant alterations in the ability of E1, E2 or TopBP1 to be recruited to the origin (Figure 6A–C). Unlike the knockdown of TopBP1, the cells treated with etoposide were arrested at G2-M, yet both conditions allow for E1–E2 DNA to be replicated (Figure 1A and Figure 5A). This demonstrates the ability of the viral proteins to bypass cellular signaling pathways to replicate DNA, but this replication is of poor quality and is highly mutagenic.

**Figure 7:**
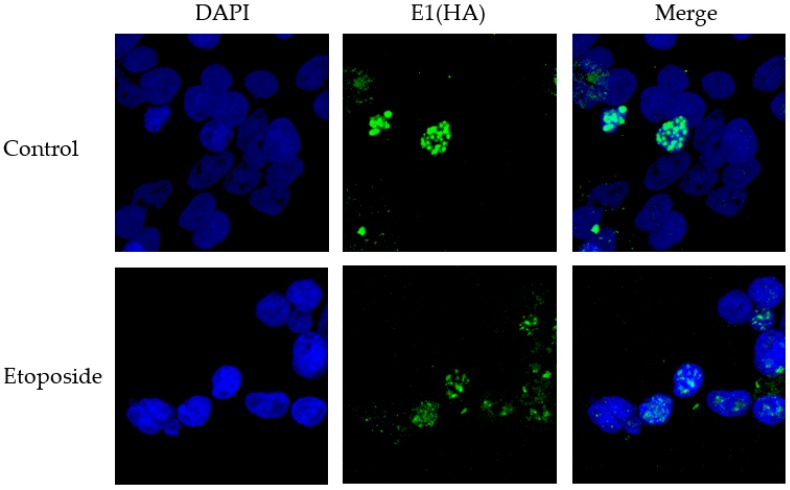
Representative images of the localization of E1 into nuclear foci in the presence and absence of etoposide. C33a cells were transfected with 1 µg pOri, 1 µg E1 (HA) and 1 µg E2. The representative images presented show typical staining patterns observed in the presence or absence of 5 µM etoposide.

Previously, our lab has shown that shRNA against TopBP1 destroys DNA replication foci induced by the expression of E1 and E2 proteins in the presence of the viral origin [32]. We sought to further elucidate the role of these replication foci to determine whether they were important in maintaining viral DNA integrity and if these foci were impacted when cells were treated with etoposide. To investigate etoposide’s impact on foci formation, C33a cells were plated onto cover slips and transfected with E1, E2 and the viral origin. Twenty four hours post-transfection, cells were grown in the presence or absence of etoposide for 48 h, and then coverslips were fixed and stained for E1 (HA). As shown in Figure 7, the addition of etoposide did not destroy the replication foci; however, there was a general trend for foci to remain much smaller (no large foci were observed in any of the etoposide-treated cells). This suggests that larger replication foci may be more efficient at maintaining genomic integrity.

## 4. Discussion

HPV can induce the DDR in infected cells to promote the viral life cycle and can recruit several DDR factors to viral replication foci [15,21,22,44]. Cellular DNA replication is controlled via the DDR pathway mediated by Ataxia telangiectasia mutated (ATM) and ATM and Rad3-related (ATR) kinases following DNA damage. Upon sensing damage, cellular DNA replication is arrested, new initiation is prevented, and DNA replication forks are stalled to allow for DNA repair [45]. As the activation of the DDR is required for the viral life cycle, it is perhaps not surprising that the virus has evolved to prevent replication shut down in the presence of this signaling. However, one consequence of this is that E1–E2-mediated DNA replication does not respond to external DNA damaging agents that are mutagenic [31]. In this report, we investigated the consequence of DNA damage signaling and genomic stress on the quality of E1–E2-mediated DNA replication.

Integration of HPV genomes into the host genome is a frequent precursor to HPV-induced carcinogenesis and likely initiated by the induction of double-stranded breaks during replication followed by non-homologous end joining (NHEJ) [14,16,18,23]. One way this breakage may occur is viral replication in the presence of DNA damaging agents. Of note, C33a cells have an arginine to cysteine mutation at position 273 of p53 resulting in its loss of function as a tumor suppressor. This mimics the virally-infected cell, which will have a non-functioning p53 due to the presence of the E6 protein targeting it for degradation. However, even though the p53 is a mutant, it is clear that the C33a cells retain a DNA damage response that arrests the cells in the G2/M phase of the cell cycle, demonstrating an appropriate response to treatment with etoposide.

The loss of TopBP1 severely compromised chromosomal integrity, as shown by the damage to chromosomes in metaphase spread cells (Figure 3). We are unable to generate cell lines with a permanent knockdown of TopBP1, although the C33a cells continued to grow for 2–3 days while accumulating chromosomal damage that would be unsustainable in the long term. TopBP1 is early embryonic lethal, supporting the essential nature of this gene [46]. Knockdown of TopBP1 did not decrease overall levels of viral replication, but resulted in enhancement of DNA damage signaling, as shown by enhanced γH2AX and Chk2 phosphorylation (Figure 2) and in mutagenic E1–E2 DNA replication (Figure 1 and Table 1). Etoposide presents the same phenotype; no change in DNA replication levels, but increased mutagenesis, indicating that the HPV replication proteins, E1 and E2, have the significant ability to replicate viral DNA under conditions of genomic stress. Under these conditions, viral replication may utilize a salvage pathway that is significantly more error prone, such as NHEJ, perpetuating the accumulation of double-stranded DNA breaks in the viral genome, which could permit integration. One difference between the results obtained with TopBP1 knockdown and etoposide treatment was the reduction in plasmid rescue into bacteria following etoposide treatment (compare Figure 1B with Figure 5B). The results suggest that there is perhaps some defect in the DNA rescued with the etoposide cells that cannot be resolved by the bacteria.

Previous [31] and current results demonstrate that etoposide, a topoisomerase IIa inhibitor that generates DSBs during the S-phase, resulting in DNA damage signaling and inhibition of DNA replication and the cell cycle, does not result in the inhibition of E1–E2 DNA replication, even though the ATR pathway is activated. Prior data correspondingly demonstrated that E1 is not a substrate for ATM/ATR *in vivo* and suggested that differential phosphorylation may allow for continued activation of E1 and E2 replication due to non-targeting of the E1 helicase following the DNA damage response [31]. Moreover, damage and arrest in the S-phase, where E2 has been shown to be stabilized [47], may also influence this replication. Importantly, responses to DSBs can be markedly influenced by cell cycle status. This presents the ideal environment for DNA damage, further replication of the HPV genome, generation of viral integration intermediates and progression to cancer [31].

DNA replication foci are highly conserved replication markers and are utilized by normal replicating cells during the S-phase [48]. Viruses, including HPV, have adapted to harness these replication foci for their own replication [14,49]. Previously, our results have shown that knockdown of TopBP1 destroys replication foci [32], so we sought to determine whether etoposide would impact these foci and try to elucidate the role replication foci might play in the quality of viral replication. While etoposide did not abolish replication foci, there was a general trend for foci to be smaller (Figure 7). Though replication foci are well documented, the role that their size, number and temporal organization play in replication is less understood. Van Veelen *et al.* previously observed that the number of foci-positive cells, foci number and foci size all change over time following dose-dependent DNA damage initiated by radiation [50]. The results presented here suggest that the larger replication foci formed by E1 and E2 may promote higher fidelity replication, as there is a general lack of these in etoposide-treated cells (Figure 7).

This report presents the novel observation that viral DNA replication by HPV 16E1 and E2 is robust, but error prone under conditions of genomic stress. Therefore, in natural infections, DNA-damaging agents would promote the ability of the virus to break by replicating in the presence of DNA damage, resulting in integration into the host genome, a potential mechanism that promotes HPV-induced tumorigenesis. Therapy designed to target HPV DNA replication should also be monitored for its effect on the quality of that replication.

## Figures and Tables

**Table 1:** Mutation frequency summary.

	shRNA control	shRNA TopBP1
Total^a^	11,210	17,428
White	59	326
MF (×10^-5^)^b^	526	1871
Rearranged^c^	46/54	54/54

^a^ Total number of colonies (blue + white) counted from all plates in triplicate; ^b^ MF, mutation frequency (calculated by white/total × 10^-5^); ^c^ rearranged/total counted, as determined by plasmid DNA that was prepared and digested with BamHI, then subjected to gel electrophoresis to highlight plasmid rearrangements. shRNA: short hairpin RNA.

**Table 2:** Densitometry of Western blots. Utilizing Li-Cor Image studio, Western blots were subjected to densitometry analysis, and data are presented as the fold increase over the relevant control.

Lane	1	2	3	4	5	6	7	8
E1	0	0	2.7	1	1	0	1.43	0.9
E2	0	0	0	1	1.5	0	0	2.9
H2AX	0	0	1	0.9	2.1	11.4	7.65	10
Chk2	1	0.3	1	0.5	0.6	0.95	0.81	0.8
pChk2	1	0	4.8	2.7	4.3	10	9.97	9.1

**Table 3:** Mutation frequency summary.

	Control	Etoposide
Total^a^	6431	1389
White	68	54
MF (×10^-5^)^b^	1057	3887
Rearranged^c^	46/54	52/54

^a^ Total number of colonies (blue + white) counted from all plates in triplicate; ^b^ MF, mutation frequency (calculated by white/total × 10^-5^); ^c^ rearranged/total counted, as determined by plasmid DNA that was prepared and digested with *Bam*HI, then subjected to gel electrophoresis to highlight plasmid rearrangements.

## Supplementary Materials

The following are available online at http://www.mdpi.com/1999-4915/8/6/175/s1, Figure S1: Representative FACS analysis of etoposide dose response.
