# Supplementary material for: DNA Damage Reduces the Quality, but Not the Quantity of Human Papillomavirus 16 E1 and E2 DNA Replication
Source: Viruses. 2016 Jun 22;8(6):175. doi: 10.3390/v8060175 (PMC4926195; doi:10.3390/v8060175)
Supplement: Supplementary file 1 [file viruses-08-00175-s001.pdf]

Viruses **2016**, *8*, 175; doi:10.3390/v8060175 www.mdpi.com/journal/viruses

Supplementary Materials: DNA Damage Reduces the Quality, but Not the Quantity of Human Papillomavirus 16 E1 and E2 DNA Replication

Molly L. Bristol, Xu Wang, Nathan W. Smith, Minkyeong P. Son, Michael R. Evans and Iain M. Morgan

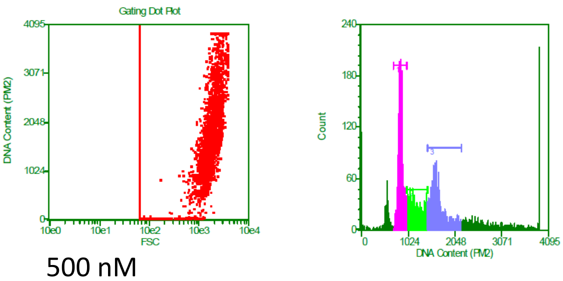

500 nM

DNA Histogram Results : Ungated

	M 1	M 2	M 3	M 4
% Positive	29.5	18.2	27.9	0.0
PM2 Mean:	868	1208	1705	0
PM2 Median:	865	1188	1654	0
PM2 %CV	6.1	10.9	10.7	0.0
Count	1474	911	1394	0

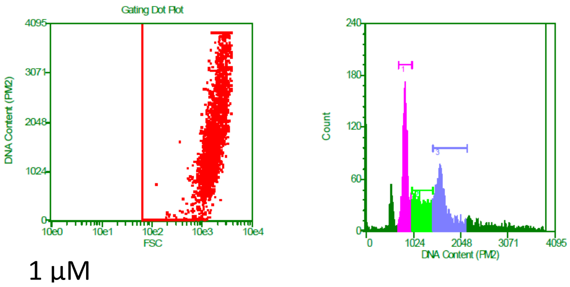

1 µM

DNA Histogram Results : Ungated

	M 1	M 2	M 3	M 4
% Positive	26.4	19.8	27.5	0.0
PM2 Mean:	858	1212	1694	0
PM2 Median:	853	1201	1641	0
PM2 %CV	6.8	10.9	10.8	0.0
Count	1322	992	1377	0

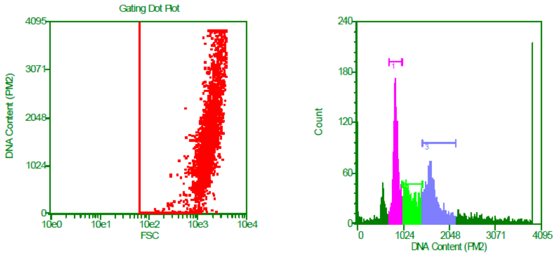

5 µM

DNA Histogram Results : Ungated

	M 1	M 2	M 3	M 4
% Positive	26.3	19.8	27.8	0.0
PM2 Mean:	868	1213	1705	0
PM2 Median:	862	1200	1659	0
PM2 %CV	6.6	10.9	10.3	0.0
Count	1317	988	1390	0

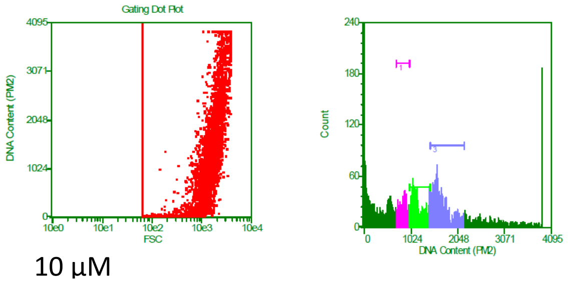

10 µM

DNA Histogram Results : Ungated

	M 1	M 2	M 3	M 4
% Positive	9.9	16.7	26.7	0.0
PM2 Mean:	855	1194	1716	0
PM2 Median:	861	1165	1661	0
PM2 %CV	9.5	11.2	11.5	0.0
Count	495	834	1333	0

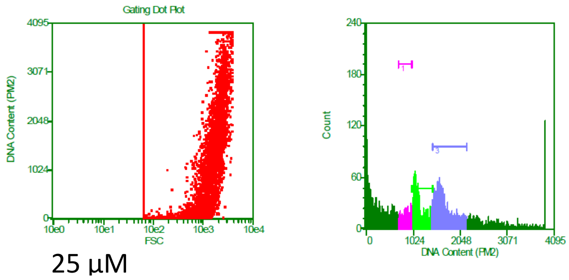

25 µM

DNA Histogram Results : Ungated

	M 1	M 2	M 3	M 4
% Positive	6.9	17.4	26.0	0.0
PM2 Mean:	862	1169	1724	0
PM2 Median:	863	1129	1668	0
PM2 %CV	10.2	10.6	11.6	0.0
Count	344	871	1302	0

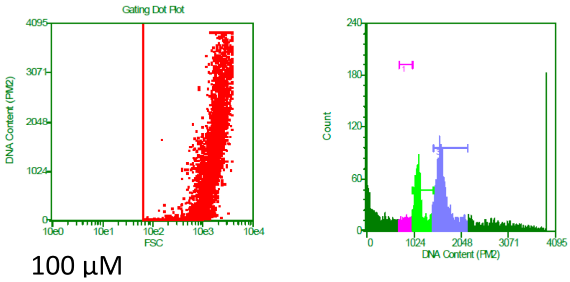

100 µM

DNA Histogram Results : Ungated

	M 1	M 2	M 3	M 4
% Positive	5.2	19.8	34.8	0.0
PM2 Mean:	855	1165	1692	0
PM2 Median:	860	1134	1638	0
PM2 %CV	10.3	9.8	10.5	0.0
Count	259	991	1741	0

Figure S1. Representative FACS analysis of etoposide dose response. C33a cells were plated; Varying doses of etoposide were added to adherent C33a cells (500 nM, 1 µM, 5 µM, 10 µM, 25 µM, 100 µM) 24 hours post plating. 48 hours after the addition of etoposide, cells were harvested and analyzed via FACS.

Viruses **2016**, *8*, 175; doi:10.3390/v8060175 www.mdpi.com/journal/viruses

© 2016 by the authors. Submitted for possible open access publication under the terms and conditions of the Creative Commons Attribution (CC-BY) license (http://creativecommons.org/licenses/by/4.0/).
